# Effects of production, fertility, health, management and conformation scores on longevity in six Swiss dairy breeds

**DOI:** 10.1016/j.vas.2026.100701

**Published:** 2026-05-17

**Authors:** Anna Bieber, Florian Hediger, Catherine Pfeifer, Urs Schnyder, Florian Leiber, Michael Walkenhorst

**Affiliations:** a Department of Livestock Sciences, Research Institute of Organic Agriculture FiBL, Ackerstrasse 113, 5070 Frick, Switzerland; b Department of Food System Sciences, Research Institute of Organic Agriculture FiBL, Ackerstrasse 113, 5070 Frick, Switzerland; c Qualitas AG, Chamerstrasse 56, 6300 Zug, Switzerland

**Keywords:** Culling risk, Dairy cattle, Longevity, Productive lifespan, Survival analysis

## Abstract

Based on herdbook data from over 2.44 million Swiss dairy cows with approximately 7.33 million lactations (01 January 1999 – 31 August 2020), culling risk factors were analyzed using Weibull proportional hazard models. Time-dependent fixed effects included fat-protein production level compared to herdmates, number of inseminations, calving interval, calving ease, lactation cell count, herd size, herd size changes, calving season, and production zone. Time-independent fixed effects comprised age at first calving, farm changes between first and last lactation, and the origin of replacement heifers, while herd-year–season was included as random effect. All traits significantly affected culling risk. Together, the investigated traits improved model fit substantially, with Maddala’s R² values ranging between 0.20 and 0.40 for productive lifespan (PL). However, most individual trait effects were small in magnitude despite statistical significance. The number of inseminations accounted for the largest proportion of PL variation across all breeds, followed, in most breeds, by the cow’s fat-protein yield relative to her herdmates. The importance of other traits varied by breed. Linear composite traits explained only a small proportion of PL variation (1.2 to 3.7 %), with a good udder score improving longevity. Cows with a high final linear score had longer PL, though its explanatory power was low (0.7 to 2.5 %), and few cows were scored at the extremes**.** The findings highlight the critical role of fertility management for longevity and the economic importance of productivity for cow survival, while emphasizing that longevity is influenced by many factors with generally small individual contributions.

## Highlights

• Fertility is a key driver of productive lifespan in Swiss dairy cows.
• Fat-protein yield is associated with longevity, highlighting its economic value.
• Production, fertility, and health traits explain 20–40 % of lifespan variation.
• Good udder and conformation scores are linked to higher survival rates.
• Longevity depends on a complex interplay of fertility, productivity, and health.

**Glossary**
AFC: age at first calving
Alp: alpine pasturing
BreedType: breed type used for the first insemination in heifers
BS: Brown Swiss
BVCH: Braunvieh Schweiz herdbook
CALV: calving ease
CI: calving interval
CS: calving season
DIM: days in milk
FatProt: cow’s fat-protein yield relative to her herdmates by year, lactation and quartiles
FeetLeg: linear composite traits for feet and leg characteristics
Frame: linear composite trait for stature and other body-related traits
FrameCap: linear composite trait for frame traits and rump traits
HO_SHB: Holstein from the swissherdbook
HO_HOS: Holstein from the Holstein Switzerland herdbook
HOS: Holstein Switzerland herdbook
HS: herd size
HYS: combined effect of herd- year- season
LRT: likelihood ratios test(s)
NINS: number of inseminations
OB: Original Braunvieh
Origin: origin of the heifer
PL: productive lifespan
RF: Red Factor
RH: Red Holstein
RHS: relative annual change of herd size
RR: relative risk or risk ratio
Rump: linear composite trait for rump properties
SCC: average lactation somatic cell count
SCS: somatic cell score
SF: Swiss Fleckvieh
SHB: swissherdbook
SI: Simmental
Teats: linear composite trait containing teat-related single traits
Udder: linear composite trait for udder related traits
Zone: production zone

## 1 Introduction

Longevity of dairy cows is decisive for sustainability of dairy production at economic, environmental and ethical level. Cows with extended productive lifespans contribute to overall efficiency by spreading rearing costs over more lactations, thus enhancing profitability (Bergeå et al., 2016). Moreover, a higher proportion of mature, higher producing cows in the herd further improves profitability (De Vries, 2020). From an environmental perspective, increasing the productive lifespan of dairy cows enhances sustainability by diluting resource inputs and emissions over a longer productive period (Wall et al., 2012; Van Middelaar et al., 2014). In addition to this dilution effect, there is evidence for improved biological efficiency in older cows, as methane emissions per unit of milk decline in cows older than 6.5 years (Grandl et al., 2016). Furthermore, longevity supports sustainable food systems by enabling the combined milk and beef production, thereby reducing overall emissions compared to specialized production systems (Probst et al., 2019; Zehetmeier et al., 2012 ). From an ethical standpoint, improved longevity is closely linked to better health and welfare, making it a desirable goal for dairy production (Ahlman et al., 2011; Oltenacu & Broom, 2010).

Despite these benefits, productive lifespan has declined in many modern dairy systems (Dallago et al., 2021; Schuster et al., 2020). Culling decisions significantly impact longevity and are influenced by both voluntary and involuntary factors. Poor fertility, health issues, and injuries mainly cause involuntary culling, while voluntary culling primarily results from low milk production (Ahlman et al., 2011; Olechnowicz et al., 2016 ; Pinedo et al., 2010; Pritchard et al., 2013; Schuster et al., 2020). External factors, such as economic conditions and the availability of genetically superior replacement heifers, also play a role (Alvåsen et al., 2018; De Vries & Marcondes, 2020).

Survival analysis is a widely used statistical approach for studying longevity in dairy cattle (Ducrocq, 2005). This method accommodates time-dependent variables and accounts for the non-linear characteristics of lifespan data. It is particularly valuable because it includes information on both living (censored) and culled (uncensored) cows, thereby maximizing available data for analysis. Swiss dairy systems differ markedly from other European contexts due to their predominantly small-scale structure (i.e. on average 29.5 ha agricultural land and 29.4 dairy cows; BLW, 2025), strong reliance on grass-based feeding (Mack et al., 2017), and use of seasonal alpine grazing (Mack et al., 2013), practiced by around 11 % of dairy farms (BLW, 2025). In addition, production is embedded in a policy framework where direct payments linked to environmental services, biodiversity, and landscape maintenance constitute a substantial share of farm income (OECD, 2025). These structural and institutional features create a highly heterogeneous production system that is distinct from more intensive, lowland-oriented European dairy sectors. Given these structural differences, although longevity has been well studied, only few older studies concern Swiss dairy cows (Vukasinovic et al., 1997; Vukasinovic et al., 2001). In addition to earlier work investigating the effect of housing transition on culling risk in Brown Swiss cows (Bielfeldt et al., 2006), recent studies have examined the economic optimal productive lifespan (Schlebusch et al., 2025) and the economic drivers of culling decisions (Gazzarin et al., 2025). However, despite these contributions, scientific evidence on the determinants of cow longevity in Swiss dairy breeds remains limited. Therefore, this study aimed to assess how production, health, fertility, and management-related traits, and composite conformation traits influence longevity. A Weibull proportional hazard model was applied to six Swiss dairy breeds to analyze these effects.

## 2 Material and methods

### 2.1 Data origin

Herdbook data for dairy cows with calving events between 01 January 1999, and 31 August 2020, were provided by the Competence Center for Informatics and Genetics of Swiss Breeding Organizations, Qualitas AG (Zug, Switzerland). The data originated from the herdbooks of Braunvieh Schweiz (BVCH, Zug, Switzerland), swissherdbook (SHB, Zollikofen, Switzerland), and Holstein Switzerland (HOS, Posieux, Switzerland). Culling reasons were additionally included from the animal tracing database ("Tierverkehrsdatenbank"), managed by Identitas AG (Bern, Switzerland).

### 2.2 Breed definitions

This study distinguished six breeds. Within the Brown Cattle population, Original Braunvieh (OB) and Brown Swiss (BS) were included. These breeds show clear genetic distance (Signer-Hasler et al., 2017) and are bred for different purposes by the breeding association. BS originated from crossbreeding U.S. Brown Swiss cattle, selected for milk production, into the OB population since the 1970s (Hagger, 2005) . Similarly, Simmental cows (SI) were crossed with U.S. Red Holstein (RH) genetics beginning in the 1970s, leading to the designation of Swiss Fleckvieh (SF) as a separate breed under the 2014 Animal Breeding Ordinance (Meier, 2020). The 2014 ordinance classified SF cows and RH cows with < 87.5 % RH blood (born before July 2008) as 100 % SF (Meier, 2020). For comparability, a consistent threshold of ≥ 87.5 % RH blood was applied for Holstein swissherdbook (HO_SHB) across all historical data, reflecting current definitions despite past changes regarding breed definitions (M. Schelling, personal communication). Average SF cows today have approximately 65 % Holstein and 35 % Simmental blood (swissherdbook, 2023).

The six breeds were classified as follows, based on breed codes (all cows), herdbook affiliation (for Holstein cows), and blood percentages (Red Holstein blood in the Red Cattle population, Brown Swiss blood in the Brown Cattle population): Brown Swiss (BS): comprising breed codes BS and Braunvieh (BV), Original Braunvieh (OB): comprising purebred OB and cow with at least 87.5 % OB blood (coded ROB), Swiss Fleckvieh (SF), Holstein from the swissherdbook (HO_SHB): comprising Red Holstein (RH), Holstein (HO), and Red Factor (RF), with at least 87.5 % HO blood, Simmental (SI), and Holstein from the Holstein Switzerland herdbook (HO_HOS).

### 2.3 Trait definitions and calculations

#### 2.3.1 Longevity, production, fertility traits, health indicators, and herd management traits

Longevity was defined as length of productive lifespan, i.e. days between first calving and culling. Functional longevity was analysed for traits related to health, fertility and management, with production effects accounted for by including fat-protein-yield relative to herdmates as a time-dependent covariate. True longevity was analysed for composite conformation traits and the final linear score, where production effects were not included in the model. .

Explanatory variables for survival analysis, including traits related to production, health, fertility and management are shown in Table 3 and in Model (1) (Section 2.5.2).

#### 2.3.2 Conformation traits

The effect of linear composite traits and the final linear score on culling risks in cows was investigated using models based on true longevity, i.e. excluding production-related effects. Conformation traits were recorded as part of the linear description provided by breeding associations, with datasets limited to the periods 2005−2020 for Holstein cows, 2008−2020 for BS and OB, and 2011−2020 for SF and SI.

Cows were evaluated for various conformation traits, each scored on a 1 to 9 scale. These scores describe the degree of expression of each trait rather than its desirability.

Individual conformation traits were grouped into linear composite traits: five for BS/OB: (frame, rump, feet & legs, udder, and teats), four for SF/ SI (frame & capacity, feet & legs, udder, and teats), and HO (frame & capacity, rump, feet & legs, and udder).

Details on trait compositions can be found in the Supplementary Material (Tables S1 - S3). Linear composite traits reflect how well a cow corresponds to classification standards. A classification score is assigned to each of the linear composite traits. The relative importance of individual traits within the breeding objectives is reflected in the weights assigned to them (e.g., see Tables S1). A final classification score (in points) was calculated by the respective breeding association by weighting these linear composite traits using breed-specific formulas that are not publicly disclosed (weights detailed in Tables S1-S3).

Final linear scores ranged from 65 to 99 points for Brown cows and < 70 to 97 points for HO, SF, and SI. This study was limited to cows classified in first lactation, where the maximum final linear score was 89; scores ≥ 90 (excellent) were reserved for second lactation onwards.

### 2.4 Data validation and editing

Data were cleaned using defined thresholds for lactation milk yield (2,000–25,000 kg), average daily milk yield (6.6–​82 kg), fat yield (60–​1,000 kg), protein yield (50–​800 kg), lactation length (up to 720 days), somatic cell count (0–​9.9 million cells/mL milk), herd size (> 2 animals), calving interval (280–​750 days), age at first calving (20–​42 months), and lactation number (1–​20). Records of cows with non-continuous lactation numbers were removed. These thresholds were based on biological plausibility and correspond to data validation standards routinely applied in Swiss national breeding value estimation. Incomplete lactations were extrapolated to a 305-day standard length using multiplication factors from the official Swiss breeding value estimation system implemented within breeding associations by Qualitas AG, following established genetic evaluation procedures to ensure comparability between animal records and evaluations (ICAR, 2022).

For short lactations (80–​269 days in milk), milk, fat, and protein yields were extrapolated using these multiplication factors. Factors varied based on five variables: production zone, lactation status, alpine pasturing, days in milk (DIM), and calving month for SHB/HOS cows; and production zone, lactation status, days open, initial daily milk yield, and calving season for BVCH cows (i.e. BS and OB).

Standard lactations of ≥ 270 DIM were corrected to a full lactation by applying multiplication factors based on DIM or days open (SHB/HOS: 270–​410 DIM in 10-days intervals, with additional factors for 430 and 460 DIM; BVCH: based on days open). All standard lactations were then corrected for age at calving within lactation class (specific age ranges applied per herdbook and lactation).

For lactations with < 80 DIM, performance data from the previous lactation were used; if unavailable, data from the subsequent lactation were used; if still unavailable, the average of all available lactations was taken. If none applied, the lactation performance was set to missing.

To ensure comparability, linear composite trait and final linear score data were included from the point of consistent availability in the linear description scheme of the respective breeding association: 2005 (HOS), 2008 (BVCH), and 2011 (SHB).

### 2.5 Survival analysis

#### 2.5.1 Trait and record types and sample sizes

Traits were categorized as either time-dependent or time-independent. Time-dependent traits were those potentially changing between lactations, while time-independent traits, such as age at first calving, remained constant throughout a cow's lifespan. Records were classified based on their status:

• Records of cows with a culling date were considered uncensored.
• Records without culling date within the study period were classified as right-censored. This also applied to records of cows with > 6 lactations, which were right- censored at the end of their sixth lactation.
• Lactation records that began before the study period (01 January 1999) were treated as left-truncated.

The validated dataset for production, fertility traits, health indicators, and herd management traits comprised over 7.33 million lactation records from more than 2.44 million dairy cows (detailed distribution in Table 1). The dataset on conformation traits included 837,660 dairy cows (detailed distribution in Table 2).

**Table 1:** Sample sizes for models on production, fertility traits, health indicators, herd management traits by breed (1999–​2020).

Breed^1^	Cows	Lactation records	Right-censored records^2^		Left-truncated records^3^		Uncensored records^4^
	n	n	n	%	n	%	n
BS	920,940	2,853,194	213,343	23.2	116,034	12.6	707,597
OB	45,629	146,352	17,173	37.6	4091	9.0	28,456
HO_HOS	262,857	709,294	17,424	6.6	20,247	7.7	245,433
HO_SHB	486,918	1,333,209	113,001	23.2	9023	1.9	373,917
SF	610,062	1,916,796	130,279	21.4	93,836	15.4	479,783
SI	120,281	372,749	32,563	27.1	13,098	10.9	87,718

1 Breeds: BS= Brown Swiss, OB= Original Braunvieh, HO_HOS= Holstein from the Holstein Switzerland herdbook, HO_SHB= Holstein from the swissherdbook, SF= Swiss Fleckvieh, SI= Simmental.

2 right-censored records: lactation records of cows still alive at the end of the study period (31.08.2020) and records of cows from the 7^th^ lactation onwards.

3 left-truncated records: Lactation records of cows that had started their lactation before the study period (01.01.1999).

4 uncensored records: lactations records of cows culled within the study period.

**Table 2:** Sample sizes for conformation traits by breed from 2008 to 2020 in Brown Swiss (BS) and Original Braunvieh (OB), from 2005 to 2020 for Holstein cows in herdbooks of Holstein Switzerland (HO_HOS) and swissherdbook (HO_SHB) and from 2011 to 2020 for Swiss Fleckvieh (SF) and Simmental (SI).

Breed	Cows	Right-censored records^1^		Uncensored records^2^
	n	n	%	n
BS	389,439	91,765	23.6	297,674
OB	22,147	8447	38.1	13,700
HO_HOS	100,160	3307	3.3	96,853
HO_SHB	212,664	64,728	30.4	147,936
SF	98,654	32,294	32.7	66,360
SI	14,596	5808	39.8	8788

1 right-censored records: records of cows still alive at the end of the study period (31.08.2020) and records of cows from the 7^th^ lactation onwards.

2 uncensored records: records of cows culled within the study period.

#### 2.5.2 Models

A proportional hazard model, assuming a piecewise Weibull distribution for the baseline hazard function (Ducrocq et al., 1988), was employed to assess the influence of various traits on the risk ratio of survival, with each breed analyzed separately. The proportional hazard assumption was assessed through graphical inspection of hazard functions over time, and no relevant deviations were observed.

Model (1) investigated traits related to production, fertility, health indicators, and herd management (listed in Table 3). The statistical model used was:

(Model 1) $$\begin{array}{c} \lambda_{\mathrm{ijkl}\mathrm{mnop}\mathrm{qrsu}\mathrm{vwx}}\left(t\right)=\lambda_{0}\left(t\right)*\exp\left\{\mathrm{FatP}\mathrm{ro}t_{i}\left(t\right)+\mathrm{NIN}S_{j}\left(t\right)+CI_{k}\left(t\right)+\mathrm{CAL}V_{l}\left(t\right)+\mathrm{SC}C_{m}\left(t\right)+HS_{n}\left(t\right)+\mathrm{RH}S_{o}\left(t\right)+CS_{p}\left(t\right)+\mathrm{Zon}e_{q}\left(t\right)+\mathrm{Al}p_{r}\left(t\right)+\mathrm{Origi}n_{s}+\mathrm{Bree}\mathrm{dTyp}e_{u}+\mathrm{AF}C_{v}+\mathrm{Chang}e_{w}+\mathrm{HY}S_{x}\left(t\right)\right\} \end{array}$$

where:

• λ(t): hazard function, representing the probability of a cow being culled at time t
• λ_0_ (t): piecewise Weibull baseline hazard function of the lactation number (1 to 6)

**Table 3:** Investigated effects used in survival analysis on production, health, fertility and herd management traits in Model (1).

Effect	Description and explanation	Number of classes	Class definition	Time -dependent	Type
FatProt	Cow’s fat-protein yield relative to her herdmates’ performance (kg) class by year and lactation number (1 or ≥2) by quartile (QT)	4	0: low:< 1^st^ QT 1: average: 1^st^ to 3^rd^ QT 2: high:> 3^rd^ QT 3: NA	yes	fixed
NINS	Number of inseminations	5	0: 1 1: 2 2: 3–​4 3: >4 4: NA	yes	fixed
CI	Calving interval (days)	5	0: ≤ 365 1: 366–​410 2: 411–​455 3: >455 4: NA	yes	fixed
CALV	Calving ease	5	1: normal 2: light help 3: difficult 4: Caesarean section 5: NA	yes	fixed
SCC^1^	Average lactation somatic cell count (in 100,000 cells/mL milk)	6	0: <70k 1: 70–​99k 2: 100–​150k 3: 151–​349k 4: >349k 5: NA	yes	fixed
HS	Herd size (number of cows)	5	0: <10 1: 10–​19 2: 20–​39 3: 40–​69 4: ≥70	yes	fixed
RHS	Relative annual change of herd size	3	0: shrinking > −20 % 1: constant between −20 and +20 % 2: increasing > + 20 %	yes	fixed
CS	Calving season	2	1: Winter (1.9.−28./29.2.) 2: Summer (1.3.−31.8.)	yes	fixed
ZONE^2^	Production zone	5	0: lowlands & hilly areas 1: mountain 1 2: mountain 2 3: mountain 3 & 4 4: NA	yes	fixed
ALP	Occurrence of alpine pasturing	3⁎	0: no 1: yes 2: NA	yes	fixed
ORIGIN	Origin of the cow, i.e. if the animal was raised on the farm or bought in	2	0: own 1: purchase	no	fixed
BREEDTYPE	Breed type used for the first insemination as heifer	5	1: dairy 2: beef 3: dual-purpose 4: other 5: NA	no	fixed
AFC	Age at first calving (months)	6	0: <24 1: 24–​26 2: 27–​29 3: 30–​32 4: 33–​35 5: >35	no	fixed
CHANGE	Occurrence of farm change between first and last lactation	2	1: yes 2: no	no	fixed
HYS	Herd⁎ year ⁎ season interaction	Log-gamma distribution		yes	random

1 SCC values were not available for HO_HOS cows.

2 The Federal Office for Agriculture records the agricultural zones and areas in digital topographical maps, which can be accessed via the following link: https://s.geo.admin.ch/6ee4f215a7.

⁎ No NA values for HO_HOS, for the respective models we had only two levels instead of three for this trait.

Time-dependent fixed effects (defined at the lactation level and referring to the current lactation):

• FatProt: effect of the cow’s fat-protein yield relative to her herdmates by year, lactation and quartiles (low, average, high),
• NINS: effect of number of inseminations,
• CI: effect of calving interval (not defined for first lactation due to its biological definition),
• CALV: effect of calving ease,
• SCC: effect of the average lactation somatic cell count (data missing for HO_HOS cows),
• HS: effect of herd size,
• RHS: effect of relative annual change of herd size (shrinking, constant, increasing),
• CS: effect of calving season (summer, winter),
• Zone: effect of the production zone (lowlands and hilly areas, mountain 1, mountain 2, mountain 3 and 4),
• Alp: effect of alpine pasturing (present, absent),

Time- independent fixed effects:

• Origin: effect of the origin of the heifer (own replacement or purchased),
• BreedType: effect of the breed used for the first insemination in heifers (dairy, beef, dual-purpose, other, missing),
• AFC: effect of age at first calving,
• Change: effect of farm change between first and last lactation.

Random effect: HYS is the combined effect of herd- year- season (log-gamma distributed).

An overview of all levels within traits is provided in Table 3.

Model (2) evaluated the effect of composite conformation traits on culling risk, with breed-specific structures:

(Model 2 for BS, OB) $\lambda_{ijklmn}\left(t\right)=\lambda_{0}\left(t\right)*\exp\{Frame_{i}+Rump_{j}+FeetLeg_{k}+Udder_{l}+Teats_{m}+HYS_{n}\}$

(Model 2 for both HO) $\lambda_{ijklm}\left(t\right)=\lambda_{0}\left(t\right)*\exp\{FrameCap_{i}+Rump_{j}+FeetLeg_{k}+Udder_{l}+HYS_{m}\}$

(Model 2 for SF, SI) $\lambda_{ijklmn}\left(t\right)=\lambda_{0}\left(t\right)*\exp\{FrameCap_{i}+FeetLeg_{k}+Udder_{l}+Teats_{m}+HYS_{n}\}$

where λ(t) and λ_0_ (t) are as defined in Model (1),

• Frame: effect of linear composite trait for stature and other body-related traits,
• Rump: effect of linear composite trait for rump properties,
• FrameCap: effect of the linear composite trait for frame traits and rump traits,
• FeetLeg: effect of the linear composite traits for feet and leg characteristics,
• Udder: effect of the linear composite trait for udder related traits, for HO also including teat- related single traits,
• Teats: effect of the linear composite trait containing teat-related single traits.

Details on the single traits included in these linear composite traits by breed are available in Supplementary Material (Tables S1 to S3).

Model 3 assessed the effect of the final linear score on culling risk, including only final linear score and HYS. The weights for linear composite traits within the final linear score for each breed are provided in Supplementary Material (Tables S1 to S3).

For Models 2 and 3, all effects in the exponential part of the models were fixed and time-independent, except HYS (random effect, log-gamma distributed).

### 2.6 Evaluation of explained variation

To assess the contribution of traits to explained variation, likelihood ratio tests (LRT) were performed (Ducrocq, 1994). These tests compared the full model with models excluding one trait at a time. The LRT test statistics followed a Chi-squared distribution, with degrees of freedom equal to the number of levels within trait minus one.

The explained variation was assessed using Maddala's R², calculated in the Survival Kit software (Mészáros et al., 2013). A larger difference in R² between the full and reduced models indicated a higher contribution of that trait to model fit, thus, to explaining differences in productive lifespan. In this context, Maddala’s R² should be interpreted as a relative measure of model fit improvement rather than a direct proportion of variance explained.

### 2.7 Software

Data validation and editing were performed using the “tidyverse” package (Wickham et al., 2019) within the statistical software R (versions 3.6.1, 4.0.3, 4.0.5, 4.2.1, 4.2.2, R Core Team, 2020).

Survival analysis, including likelihood ratio tests, was performed separately for each breed using the Survival Kit software (version 6.12, Mészáros et al., 2013). Three models were applied per breed: Model (1) included traits on production, fertility, health indicators, and herd management traits; Model (2) included linear composite traits, and Model 3 included the final linear scores.

A P-value of < 0.05 was interpreted as the threshold for statistical significance in all statistical analyses.

## 3 Results

### 3.1 Survival analysis on production, fertility traits, health indicators, and herd management traits

This study investigated the influence of various traits on the risk of culling across six Swiss dairy breeds. All analyzed traits significantly affected culling risk; however, most effects were small in magnitude, while collectively showing moderate explanatory power, with Maddala’s R^2^ values ranging between 0.20 and 0.40 for productive lifespan. Results for the cow’s fat-protein yield relative to her herdmates, number of inseminations, calving interval and somatic cell count are presented in tables within the main text, while results for other traits are provided in the Supplementary material.

#### 3.1.1 Fat-protein yield relative to the cow’s herdmates

Cows classified as high producers had a significantly lower culling risk compared to average producers (reference level) in all breeds but HO_HOS, with relative risk (RR) values ranging from 0.78 (OB) to 0.93 (HO_SHB, SF). Conversely, lower producing cows had an increased culling risk, particularly in OB (RR = 1.22), BS (RR = 1.19), and SI (RR = 1.14) (Table 4). Cows with missing FatProt data showed the highest culling risks across all breeds, with RR values peaking at 6.63 in SF and 4.87 in HO_SHB (Table 4).

**Table 4:** Relative risk of culling for the production trait within- herd fat-protein performance (FatProt) in six Swiss dairy breeds.

FatProt class^1^	Relative risk (RR) of culling by breed^2^ and number of uncensored failures (n)											
	BS		OB		HO_HOS		HO_SHB		SF		SI	
	RR	n	RR	n	RR	n	RR	n	RR	n	RR	n
low	1.19	244,097	1.22	9474	0.97	77,204	1.09	121,219	1.09	157,571	1.14	30,944
average^3^	1.00	285,889	1.00	11,807	1.00	104,339	1.00	150,369	1.00	195,409	1.00	34,311
high	0.80	122,012	0.78	4656	0.86	51,901	0.93	72,939	0.93	97,344	0.86	14,543
NA	3.59	55,599	3.42	2519	3.66	11,989	4.87	29,390	6.63	29,459	3.38	7920

1 Cow’s fat-protein yield relative to her herdmates’ performance was classified into four classes by quartiles within herd, year (1999–2020), and lactation status (primiparous or multiparous). low: cows in the first quartile were classified as low producers, average: cows between the first and third quartiles were classified as average producers, high: cows above the third quartile were classified as high producers, NA: missing values.

2 Breed: BS= Brown Swiss, OB= Original Braunvieh, HO_HOS= Holstein from the Holstein Switzerland herdbook, HO_SHB= Holstein from the swissherdbook, SF= Swiss Fleckvieh, SI= Simmental.

3 reference class; all risk levels differed significantly from the reference class according to the Chi-square statistic at a level of P ≤ 0.0001. RR > 1.0 indicates an increased culling risk compared to the reference, while RR < 0.1 indicates a reduced culling risk.

#### 3.1.2 Number of inseminations

The number of inseminations was a major determinant of culling risk and accounted for the largest proportion of PL variation across all breeds. Generally, an increasing number of inseminations led to a higher culling risk in most breeds (Table 5). Compared to cows with one insemination (reference level), cows with four or more inseminations had a culling risk ranging between 2.53 and 3.55 for HO_SHB, SI, SF, BS and OB, but only 1.93 in HO_HOS (Table 5).

**Table 5:** Relative risk of culling for the fertility-related traits number of inseminations (NINS) and calving interval (CI) in six Swiss dairy breeds.

Traits and class definitions^1^	Relative risk (RR) of culling by breed^2^ and number of uncensored failures (n)											
	BS		OB		HO_HOS		HO_SHB		SF		SI	
	RR	n	RR	n	RR	n	RR	n	RR	n	RR	n
NINS												
1^3^	1.00	164,187	1.00	7321	1.00	52,364	1.00	84,367	1.00	126,603	1.00	23,606
2	1.44^⁎⁎⁎⁎^	98,108	1.26^⁎⁎⁎⁎^	3731	0.81^⁎⁎⁎⁎^	35,187	1.21^⁎⁎⁎⁎^	51,865	1.25^⁎⁎⁎⁎^	71,514	1.00	11,017
3–​4	2.08^⁎⁎⁎⁎^	91,771	1.75^⁎⁎⁎⁎^	3104	1.03^⁎⁎⁎^	35,345	1.65^⁎⁎⁎⁎^	52,536	1.71^⁎⁎⁎⁎^	60,551	1.22^⁎⁎⁎⁎^	7652
>4	2.74^⁎⁎⁎⁎^	39,930	2.38^⁎⁎⁎⁎^	1109	1.12^⁎⁎⁎⁎^	18,075	2.31^⁎⁎⁎⁎^	22,364	2.49^⁎⁎⁎⁎^	18,965	1.13^⁎⁎⁎⁎^	1984
NA	3.51^⁎⁎⁎⁎^	313,601	3.55^⁎⁎⁎⁎^	13,191	1.93^⁎⁎⁎⁎^	104,462	2.54^⁎⁎⁎⁎^	162,785	2.60^⁎⁎⁎⁎^	202,150	2.60^⁎⁎⁎⁎^	43,459
CI												
≤ 365^3^	1.00	170,456	1.00	7208	1.00	49,590	1.00	85,717	1.00	147,807	1.00	28,226
366–​410	0.93^⁎⁎⁎⁎^	168,483	0.91^⁎⁎⁎⁎^	6967	0.82^⁎⁎⁎⁎^	49,521	0.91^⁎⁎⁎⁎^	75,254	0.87^⁎⁎⁎⁎^	121,111	0.89^⁎⁎⁎⁎^	21,221
411–​455	0.94^⁎⁎⁎⁎^	88,408	0.91^⁎⁎⁎⁎^	3338	0.81^⁎⁎⁎⁎^	31,790	0.89^⁎⁎⁎⁎^	44,026	0.83^⁎⁎⁎⁎^	50,936	0.88^⁎⁎⁎⁎^	6949
>455	0.96^⁎⁎⁎⁎^	102,483	0.92^⁎⁎⁎^	3461	0.79^⁎⁎⁎⁎^	42,966	0.87^⁎⁎⁎⁎^	50,029	0.80^⁎⁎⁎⁎^	43,966	0.91^⁎⁎⁎⁎^	4909
NA	0.30^⁎⁎⁎⁎^	177,767	0.85	7482	1.12	71,566	0.89^⁎⁎^	118,891	0.15^⁎⁎⁎⁎^	124,963	0.94	26,413

^1^ NA= missing value., ^2^ Breed: BS= Brown Swiss, OB= Original Braunvieh, HO_HOS= Holstein from the Holstein Switzerland herdbook, HO_SHB= Holstein from the swissherdbook, SF= Swiss Fleckvieh, SI= Simmental., ^3^ reference class; risk levels differ significantly from the reference class according to the Chi-square statistic as follows: ^⁎⁎^P ≤ 0.01, ^⁎⁎⁎^ P ≤ 0.001, ^⁎⁎⁎⁎^ P ≤ 0.0001. RR > 1.0 indicates an increased culling risk compared to the reference, while RR < 0.1 indicates a reduced culling risk.

#### 3.1.3 Calving interval

Cows with a longer calving interval (CI) generally had a lower risk of culling across most breeds (Table 5). Compared to cows with a CI of ≤ 365 days (reference level, RR = 1.00), those with a CI of 366–410 days had a significantly reduced culling risk, ranging from 0.82 (HO_HOS) to 0.93 (BS). A further decrease in risk was observed for cows with a CI of 411–455 days, with relative risks as low as 0.79 in HO_HOS and 0.80 in SF.

#### 3.1.4 Calving ease

For calving ease (CALV), the reference level was set at unassisted calving and presented in Table S4. Cows requiring light assistance during calving had an equal or only slightly higher culling risk than those with unassisted calving (reference level), with RR values ranging from 1.00 (OB, HO_HOS, SI) to 1.08 (BS) (Table S4). Difficult births increased the risk, particularly in OB (RR = 1.47). However, cows undergoing Caesarean sections showed no significant difference in culling risk compared to unassisted calving (RR ≈ 1.00 across breeds).

#### 3.1.5 Somatic cell count

Results on the risk of culling associated with SCC are shown in Table 6. In most breeds, an increase in SCC (70,000–​99,000 cells/mL) showed minor deviations from the reference (< 70,000 cells/mL), with relative risks ranging from 0.97 to 1.04, though some were statistically significant. High SCC levels (> 349,000 cells/mL) showed the highest relative culling risk across all breeds, with RR values ranging from 1.20 (SI) to 1.54 (HO_SHB).

**Table 6:** Relative risk of culling for the udder health-related trait average lactation somatic cell count (SCC, in 100,000 cells/mL milk) in five Swiss dairy breeds.

Traits and class defintions^1^	Relative risk (RR) of culling by breed^2^ and number of uncensored failures (n)									
	BS		OB		HO_SHB		SF		SI	
	RR	n	RR	n	RR	n	RR	n	RR	n
SCC										
<70k ^3^	1.00	321,117	1.00	15,815	1.00	189,868	1.00	258,569	1.00	56,007
70–​99k	0.98^⁎⁎⁎⁎^	90,533	1.02	3062	1.04^⁎⁎⁎⁎^	46,427	0.99*	56,582	0.97^⁎⁎^	8652
100–​150k	1.03^⁎⁎⁎⁎^	88,575	1.13^⁎⁎⁎⁎^	2899	1.09^⁎⁎⁎⁎^	42,209	1.05^⁎⁎⁎⁎^	51,529	1.02	7507
151–​349k	1.14^⁎⁎⁎⁎^	120,431	1.20^⁎⁎⁎⁎^	3870	1.21^⁎⁎⁎⁎^	53,114	1.17^⁎⁎⁎⁎^	65,878	1.10^⁎⁎⁎⁎^	9390
>349k	1.32^⁎⁎⁎⁎^	81,672	1.45^⁎⁎⁎⁎^	2433	1.54^⁎⁎⁎⁎^	41,613	1.50^⁎⁎⁎⁎^	46,835	1.20^⁎⁎⁎⁎^	6094
NA	0.98	5269	1.10	377	0.94	686	0.95	390	1.00	68

^1^ NA= missing value, ^2^ Breed: BS= Brown Swiss, OB= Original Braunvieh, HO_SHB= Holstein from the swissherdbook, SF= Swiss Fleckvieh, SI= Simmental, ^3^ reference class; risk levels differ significantly from the reference class according to the Chi-square statistic as follows: *P ≤ 0.05, ^⁎⁎^ P ≤ 0.01, ^⁎⁎⁎⁎^ P ≤ 0.0001. RR > 1.0 indicates an increased culling risk compared to the reference, while RR < 0.1 indicates a reduced culling risk.

#### 3.1.6 Herd size and herd size changes

Relative risk linked to herd size (HS) categories (reference level: 20–​39 cows) and relative change in herd size (RHS, reference level: constant herd size with herd size change between −20 % and +20 %) are shown in Supplementary Table S5. Cows in smaller herds (< 10 cows) generally had a lower risk of culling, particularly in HO_SHB (RR = 0.59) and BS (RR = 0.84). Cows managed in larger herds (≥ 70 cows) showed an increased risk in HO_HOS (RR = 1.93) but decreased risk in most other breeds.

A relative decrease in herd size (by at least −20 %) was associated with a lower risk of culling in most breeds, particularly in OB (RR = 0.69) and HO_SHB (RR = 0.76). A relative increase in herd size (by at least +20 %) had a minor impact, with RR values close to 1.00, except for slight reductions in HO_HOS and SF (Table S5).

#### 3.1.7 Calving season

Cows calving in summer had a significantly lower risk of culling across all breeds, compared to the reference level, i.e. winter calving (Table S6). The largest reduction of RR was observed in SI (RR = 0.87) and HO_HOS (RR = 0.90). Other breeds also showed a moderate decrease in culling risk, with RR values ranging from 0.93 (OB, SF) to 0.96 (BS, HO_SHB) (Table S6).

#### 3.1.8 Production zone and alpine pasturing

Risk ratios related to production zone and alpine pasturing are shown in Table S7. Cows in lower mountain areas had a similar culling risk as those in lowlands/hilly areas (reference level), with RR values close to 1.00. In higher mountain areas, the RR slightly increased in some breeds (e.g., BS: 1.05, OB: 1.02). SF and SI showed a slight reduction in culling risk in higher mountain areas (RR = 0.96 and 0.95, respectively).

Cows sent to alpine pastures had a significantly lower culling risk across all breeds (Table S7), particularly in SF (RR = 0.62), SI (RR = 0.58), BS (RR = 0.65), and OB (RR = 0.65), while HO_SHB only showed a moderate reduction in culling risk (RR = 0.76), and HO_HOS had only a slight decrease (RR = 0.94).

#### 3.1.9 Farm changes between first and last lactation and origin of heifers

Cows without farm change between first and last lactation were the reference level. Cows that changed farms between their first and last lactation had a significantly higher risk of culling in most breeds, particularly in OB (RR = 1.46) and HO_SHB (RR = 1.32), while BS and SI both showed a RR of 1.25. SF also showed a slight increase (RR = 1.09), whereas HO_HOS had a notably lower risk (RR = 0.47) (Table S8).

For the trait ORIGIN heifers raised on the same farm where they were born served as the reference (RR = 1.00) (Table S8). Purchased heifers had a lower risk of culling across all breeds, with RR values ranging from 0.86 (OB, SI) to 0.94 (HO_HOS) when compared to own replacement cows, with the strongest reduction observed in OB and SI (both RR = 0.86), followed by BS (RR = 0.88) and SF (RR = 0.89).

#### 3.1.10 Breed type used for insemination in heifers

The relative risk of culling varied depending on the breed type used for the first insemination as a heifer (BREEDTYPE) across the six Swiss dairy breeds (Table S9). The reference level differed between dairy breeds (BS, HO_HOS, HO_SHB, SF) and dual-purpose breeds (OB, SI). Dairy breeds were compared to heifers inseminated with dairy bulls, while dual-purpose breeds were compared to heifers inseminated with dual-purpose bulls.

In dairy breeds (BS, HO_HOS, HO_SHB, SF), heifers inseminated with beef breed bulls had a slightly reduced risk of culling compared to those inseminated with dairy breed bulls. The reduction was most pronounced in SF (RR = 0.88), followed by HO_SHB (RR = 0.92) and BS (RR = 0.95). In contrast, HO_HOS showed no significant difference (RR = 0.99). Dairy heifers inseminated with dual-purpose breed bulls also had a slightly lower culling risk in some dairy breeds. BS (RR = 0.93), SF (RR = 0.90), and HO_SHB (RR = 0.95) showed minor reductions in culling risk, while again HO_HOS showed no difference (RR = 1.00). Dairy heifers inseminated with bulls from other breeds showed no clear trends, though slight reductions in culling risk were observed for BS (RR = 0.92) and SF (RR = 0.93).

Dual-purpose breed heifers (OB, SI) inseminated with dairy breed bulls had a slightly increased risk of culling compared to those inseminated with dual-purpose bulls in SI (RR = 1.09), while risk increase in OB was not statistically significant (RR = 1.10). Dual-purpose heifers inseminated with beef breed bulls had a slightly lower risk of culling in both OB (RR = 0.94) and SI (RR = 0.97). Dual-purpose heifers inseminated with bulls from other breeds showed no significant differences in culling risk (OB: RR = 0.92, SI: RR = 0.99).

#### 3.1.11 Age at first calving

Results for age at first calving (AFC) indicate that deviations from the reference (27–​29 months) influenced the relative risk of culling, with very late AFC generally associated with increased risk (Table S10). Early AFC (< 24 months) was associated with a significantly lower risk of culling in most breeds, except for HO_HOS, where it was slightly increased (RR = 1.02). The most substantial reduction in culling risk was observed in OB (RR = 0.76), followed by BS (RR = 0.85) and HO_SHB (RR = 0.89). SI (RR = 0.93) and SF (RR = 0.91) also showed reduced risk. Heifers with an AFC between 24 and 26 months showed a lower risk of culling compared to the reference category (27–​29 months) in all breeds. The greatest reduction was observed in OB (RR = 0.81), followed by HO_HOS (RR = 0.92) and HO_SHB (RR = 0.94). The effect was similar for BS (RR = 0.97), SF (RR = 0.95), and SI (RR = 0.95). A slight increase in culling risk was observed in some breeds with AFC between 30 and 32 months, but the effect was not highly pronounced nor consistent across breeds: SI had the highest RR (1.03), followed by BS (RR = 1.02), while HO_SHB, OB and HO_HOS showed slightly reduced or no difference in culling risk (RR = 0.98, 0.89 and 0.96, respectively). A higher risk of culling was observed in most breeds for AFC between 33 and 35 months, with the highest increase in SI, BS, and SF (RR = 1.05, 1.04 and 1.02, respectively), while OB showed no significant difference (RR = 0.97). Very late first calving (> 35 months) consistently showed the highest risk of culling across all breeds, where the highest risk was observed in BS, SI, and HO_SHB (RR = 1.08, 1.04 and 1.03, respectively). OB and HO_HOS showed no significant difference compared to the reference group.

### 3.2 Survival analysis of conformation traits

Relative risks associated with linear composite traits are presented in Table 7. Across all breeds, udder consistently ranked as the most influential composite conformation trait, with the highest explained variation for productive lifespan. The exclusion of udder traits from the model resulted in the largest reduction in R² values.

**Table 7:** Ranking of the linear composite traits of the linear description according to explained variation (R^2^ of Maddala derived from likelihood ratio tests) for productive lifespan in Swiss dairy breeds.

Breed	Ranking	Linear composite trait^1^	R^2^ of Maddala the model with for all linear composite traits	R^2^ of Maddala after exclusion of a linear composite trait
Brown Swiss	1	Udder	0.0259	0.0148
	2	Rump		0.0240
	3	Feet and legs		0.0242
	4	Teats		0.0247
	5	Frame		0.0254
Original Braunvieh	1	Udder	0.0420	0.0278
	2	Feet and legs		0.0391
	3	Teats		0.0395
	4	Rump		0.0403
	5	Frame		0.0407
Holstein- Holstein CH	1	Udder	0.0115	0.0057
	2	Feet and legs		0.0082
	3	Frame and Capacity		0.0109
	4	Rump		0.0110
Holstein-swissherdbook	1	Udder	0.0175	0.0076
	2	Feet and legs		0.0135
	3	Frame and Capacity		0.0162
	4	Rump		0.0168
Swiss Fleckvieh	1	Udder	0.0163	0.0094
	2	Feet and legs		0.0134
	3	Teats		0.0152
	4	Frame and Capacity		0.0162
Simmental	1	Udder	0.0365	0.0239
	2	Teats		0.0329
	3	Feet and legs		0.0349
	4	Frame and Capacity		0.0360

1 more information on the linear composite traits is provided in Supplementary Tables S1-S3.

While the exact ranking varied slightly among breeds, feet and legs, teats, rump, and frame (or frame and capacity) also contributed to predicting productive lifespan.

Results on the relative risks linked to the final linear score are presented in Table 8 and were similar across different dairy breeds. Poor-scoring cows had relative risks ranging from 1.82 (SF) to 3.48 (BS), indicating a markedly increased likelihood of being culled. In contrast, cows with a very good final linear score had relative risks well below 1.0 (range: 0.30 in SI to 0.85 in HO_HOS).

**Table 8:** Relative risk of culling by final linear score and explained variation (R2 of Maddala) in six Swiss dairy breeds.

Breed	Score^1^	Risk Ratio	Number of uncensored failures	R^2^ of Maddala
Braunvieh	1	3.48^⁎⁎⁎⁎^	80	0.0128
	2	2.22^⁎⁎⁎⁎^	3380	
	3	1.28^⁎⁎⁎⁎^	77,800	
	4^2^	1.00	212,797	
	5	0.65^⁎⁎⁎⁎^	3617	
Original Braunvieh	1	1.03	3	0.0137
	2	2.04^⁎⁎⁎⁎^	168	
	3	1.31^⁎⁎⁎⁎^	3651	
	4^2^	1.00	9634	
	5	0.67^⁎⁎⁎⁎^	244	
Holstein- Holstein Switzerland	1	2.14^⁎⁎⁎⁎^	298	0.007
	2	1.51^⁎⁎⁎⁎^	4270	
	3	1.14^⁎⁎⁎⁎^	41,249	
	4^2^	1.00	50,308	
	5	0.85^⁎⁎⁎^	728	
Holstein-swissherdbook	1	1.91^⁎⁎⁎⁎^	682	0.0083
	2	1.28^⁎⁎⁎⁎^	8789	
	3^2^	1.00	71,278	
	4	0.86^⁎⁎⁎⁎^	66,783	
	5	0.50^⁎⁎⁎⁎^	404	
Swiss Fleckvieh	1	1.82^⁎⁎⁎⁎^	246	0.0098
	2	1.23^⁎⁎⁎⁎^	3644	
	3^2^	1.00	32,547	
	4	0.82^⁎⁎⁎⁎^	29,778	
	5	0.40^⁎⁎⁎⁎^	145	
Simmental	1	2.04^⁎⁎⁎⁎^	57	0.0249
	2	1.30^⁎⁎⁎⁎^	603	
	3^2^	1.00	4085	
	4	0.72^⁎⁎⁎⁎^	3978	
	5	0.30^⁎⁎⁎⁎^	65	

^1^ Score: 1= < 70 points, P-poor, 2 = 75–​79 points, F-fair, 3 = 75–​79 points: G-good, 4 = 80–​84 points, GP- good plus, 5 = 85–​89 points, VG- very good, ^2^ reference class; risk levels differ significantly from the reference class according to the Chi-square statistic as follows: ^⁎⁎⁎^P ≤ 0.001, ^⁎⁎⁎⁎^ P ≤ 0.0001. Risk ratio (RR) > 1.0 indicates an increased culling risk compared to the reference, while RR < 0.1 indicates a reduced culling risk.

### 3.3 Share of explained variation

We used likelihood ratio tests to assess the relevance of each trait in explaining the variation in productive lifespan by comparing the full model to a model in which one explanatory trait was removed at a time for each breed separately. All investigated traits had a highly significant effect on longevity (P < 0.0001).

In Model (1), the total explained variation, expressed as Maddala’s R², ranged between 21 and 40 % (Table 9). Ranking on the contribution of single traits was based on the relative changes observed in Maddala’s R² when a trait was removed from the full model (Table 9).

**Table 9:** Explained variation measured as Maddala’s R^2^ by traits included in Model (1) of the Survival analysis across six Swiss dairy breeds.

	Breed^1^ and total Maddala’s R^2^ (%)					
	BS 0.3968	OB 0.2801	HO_HOS 0.2069	HO_SHB 0.2295	SF 0.2549	SI 0.2415
Trait^2^	Maddala’s R^2^ after removal of the respective trait					
FatProt	0.3672	0.2607	0.1298	0.1837	0.1710	0.2158
NINS	0.2736	0.1700	0.1233	0.1474	0.1590	0.0670
CI	0.3864	0.2882	0.1745	0.2267	0.2330	0.2478
CALV	0.3936	0.2713	0.2069	0.2278	0.2544	0.2267
SCC^3^	0.3930	0.2702		0.2209	0.2465	0.1486
HS	0.3942	0.2867	0.1624	0.2213	0.2471	0.2563
RHS	0.3945	0.2821	0.1657	0.2249	0.2492	0.2580
CS	0.3913	0.2854	0.1430	0.2226	0.2448	0.2540
ZONE^4^	0.3839	0.2754	0.1302	0.2067	0.2251	0.2382
ALP	0.3859	0.2746	0.1560	0.2257	0.2174	0.2356
ORIGIN	0.3922	0.2849	0.1439	0.2209	0.2425	0.2559
BREEDTYPE	0.3913	0.2854	0.1437	0.2200	0.2410	0.2548
AFC	0.3919	0.2845	0.1442	0.2193	0.2134	0.2551
CHANGE	0.3917	0.2818	0.1322	0.2169	0.2431	0.2562

Total Maddala’s R^2^ represents the proportion of variation that is explained by Model (1), including all listed variables. Maddala’s R^2^ after removal represents the proportion of variation explained by the reduced model after excluding the respective trait. A larger difference between these values indicates a greater contribution of the respective trait to explaining variation in longevity.

1 Breed: BS= Brown Swiss, OB= Original Braunvieh, HO_HOS= Holstein from the Holstein Switzerland herdbook, HO_SHB= Holstein from the swissherdbook, SF= Swiss Fleckvieh, SI= Simmental.

2 Trait: FatProt= Cow’s fat-protein yield relative to her herdmates’ performance (kg) class by year and lactation number (1 or ≥2) by quartiles (QT), NINS= Number of inseminations, CI= calving interval (days), CALV= calving ease, SCC= Average lactation somatic cell count (in 100,000 cells/mL milk), HS= herd size (number of cows), RHS= Relative annual change of herd size, CS= calving season, ZONE= production zone, ALP= occurrence of alpine pasturing, ORIGIN= Origin of the cow, i.e. if the animal was raised on the farm or bought in, BREEDTYPE= Breed type used for the first insemination as heifer, AFC= Age at first calving (months), CHANGE= Occurrence of farm change between first and last lactation.

3 SCC values were not available for HO_HOS cows.

4 The Federal Office for Agriculture records the agricultural zones and areas in digital topographical maps, which can be accessed via the following link: https://s.geo.admin.ch/6ee4f215a7.

Among all breeds, the number of inseminations contributed the most to explaining productive lifespan variation, followed by the cow’s fat-protein yield relative to her herdmates in all breeds. The third most influential trait varied by breed and had a smaller effect overall. Production zone was the third most relevant trait in OB, SF, HO_HOS, and HO_SHB. In BS, alpine pasturing ranked third. In SI, farm changes between first until last lactation ranked third. From the third or fourth rank onward, the proportion of explained variation decreased considerably.

For linear composite traits, the total explained variation in Model (2) ranged from 1.2 % to 4.2 % (Table 7).

Finally, the final linear score of linear description explained only 0.7 % to 2.5 % of the variation in productive lifespan (Table 8).

## 4 Discussion

### 4.1 Fat-protein yield relative to the cow’s herdmates

Our finding that low-producing cows faced a higher risk of culling, while high-producing cows were generally retained, aligns with studies that have investigated this trait across different breeds and countries (e.g., De Vries & Marcondes, 2020; Pinedo et al., 2010). As the quantity of milk sold is the backbone of the economic viability of dairy farms, this result is expected. A Swiss study on Brown Swiss cows found that cows producing < 90 % of the yield of their herdmates with the same lactation number had the highest culling risk, whereas high-performing cows (those producing > 110 % of the yield of their herdmates) had a considerably lower culling risk, with a risk ratio of 0.64 (Bielfeldt et al., 2006). Another Swiss study confirmed these findings for both Holstein and Brown Swiss cows, reporting even more pronounced increases in culling risk. Specifically, Holstein cows yielding < 80 % compared to herdmates had a three to four times higher risk of being culled (Vukasinovic et al., 2001). While some studies suggest that the increased culling risk is primarily driven by low production levels, with limited additional effects of above-average production (Rajala-Schultz & Gröhn, 1999; Strandberg & Roxström, 2000), our results indicate that both low and high production levels were associated with changes in culling risk. At the same time, high milk production is often associated with increased metabolic and reproductive challenges, which may negatively affect fertility (e.g., Berry et al., 2014; Pritchard et al., 2013). Our results indicate that high production alone is not sufficient to ensure retention, as fertility-related traits, particularly the number of inseminations, were strong determinants of culling risk.

### 4.2 Fertility traits

Fertility problems are widely recognized as the most common reason for culling in Swiss breeds (Bieber et al., 2026). Therefore, it is unsurprising that we observed a significantly increased culling risk associated with a higher number of inseminations across most breeds. The strong association between the number of inseminations and culling risk likely reflects both underlying biological limitations (e.g. reduced conception success) and management-related factors such as oestrus detection efficiency and culling decisions.

Interestingly, a longer calving interval (CI) was generally associated with a lower risk of culling across all breeds, with the greatest reduction observed in HO_HOS. However, this result warrants careful interpretation, as a longer calving interval is inherently associated with a longer productive lifespan. In addition, because the number of inseminations is included in the model, the effect of calving interval reflects differences at a given level of reproductive success. An extended calving interval may reflect either fertility problems or deliberate management decisions, such as extending lactation length through delayed insemination. On the one hand, in grassland-based production systems with seasonal calving, there is greater pressure on cows to conceive quickly compared to indoor-housed systems with continuous calving. On the other hand, intentionally prolonging the calving interval is increasingly regarded as a sensible management strategy in higher-yielding breeds (such as HO_HOS), as it reduces the frequency of critical transitions such as dry-off, calving, and the start of a new lactation (van Knegsel et al., 2022). This approach has been associated with improved productive lifespan in some studies (Han et al., 2022; Owusu-Sekyere et al., 2023). Additionally, the variation observed in missing CI data should be noted. First-lactation cows do not yet have calving interval information, and estimates for the missing data level are biased by the higher culling rates typically observed in first-lactation cows (Bieber et al., 2026).

The need for assisted calving is another factor significantly associated with increased culling risk (Olechnowicz et al., 2016). In our analysis, cows requiring assistance during birth had a slightly higher culling risk, particularly in OB, where difficult calving posed the highest risk. However, Caesarean sections did not significantly affect culling risk, likely due to their low incidence across all breeds. These findings align with an Italian study that reported higher culling risk until 30 DIM for Holstein cows with assisted calving, as well as lower pregnancy rates until 150 DIM and reduced milk yield (Probo et al., 2022). Additionally, Pritchard et al. (2013) emphasized that cows with high longevity are characterized by better reproductive performance, including shorter calving interval and fewer inseminations required to conceive, thereby highlighting the importance of reproductive efficiency in promoting cow survival and longevity.

### 4.3 Somatic cell count

Udder health problems are the second most common reason for culling in Swiss dairy breeds (Bieber et al., 2026) and have a significant negative effect on both animal welfare and economic performance due to reduced milk yield. In this context, our finding that higher SCC levels are strongly associated with an increased risk of culling across all breeds, with the greatest impact observed in HO_SHB and OB breeds, is consistent with previous research. Cows with elevated SCC are more likely to be culled, as demonstrated in several studies.

For example, Archer et al. (2013) reported a 5 % increase in the odds of culling per unit increase in log-transformed SCC during the first lactation in Irish dairy cows. Abfalter et al. (2016) found that German Holstein cows with exceptional longevity (> 9 lactations) had lower somatic cell score (SCS) levels and higher relative breeding values for somatic cell count compared with their herdmates. Similarly, Caraviello et al. (2005) reported that cows with average SCC exceeding 700,000 cells/mL had a culling risk 2.2 to 4 times higher than cows with SCC levels between 200,000 and 250,000 cells/mL. A Canadian study by Sewalem et al. (2006) revealed that cows with SCS above the specific breed average had a substantially increased relative risk of culling: Holstein, Ayrshire, and Jersey cows in the highest SCS categories were 4.95, 6.73, and 6.62 times more likely, respectively, to be culled compared to cows with average SCS levels. Likewise, Samoré et al. (2003) reported that Italian Holstein cows in the highest SCS class had more than triple the culling risk compared to those in the lowest class.

While our findings generally align with these studies, the risk increase observed in Switzerland was less pronounced. This discrepancy may be attributed to the generally lower SCC levels in Swiss herds compared to those reported in other countries.

### 4.4 Herd size and herd size changes

Our analysis revealed that herd size and changes in herd size were associated with culling risk, with differences across breeds. In general, smaller herds and those with decreasing sizes tended to show lower culling risks. However, these associations should be interpreted with caution, as causality cannot be inferred from the present analysis, and the observed relationships may be influenced by unmeasured confounding factors. One possible explanation is that smaller herds may allow for more individual cow attention, which could contribute to improved health and longevity.

Switzerland’s relatively small average herd sizes make direct comparisons with other countries challenging. For example, a Dutch study reported that cows in smaller herds (< 50 cows) had an average age at culling that was 130 days higher than cows in larger herds (> 201 cows) (Han et al., 2022). In contrast, a Polish study found that herd size had only a minor effect on age at culling (Adamczyk et al., 2017). Additionally, a Swedish study reported that cows in larger or expanding herds were 50 % less likely to achieve a long productive lifespan (Strandberg & Emanuelson, 2016). Overall, these findings suggest that herd size and herd size development are associated with culling risk, although the specific effects may vary by country and production system.

### 4.5 Calving season, production zone, alpine pasturing

The lower culling risk observed for cows calving from spring to summer across all breeds, with the strongest effects in SI and HO_HOS cows, can likely be attributed to improved nutrient conditions during these months in Switzerland. Grassland-based production is widespread in Switzerland, with 87 % of dairy farms receiving direct payments for pasture-based production (Mack et al., 2017). Seasonal effects on longevity have been reported by other studies, but direct comparisons are challenging due to differences in climatic conditions and seasonal classifications, i.e. the division into four seasons (e.g., Rakhshani Nejad et al., 2021). Additionally, reproductive performance in grassland-based systems could be more affected by heat stress during summer (Dallago et al., 2021).

The classification into production zones is unique to Switzerland, which may explain the lack of references regarding survival probabilities based on this factor. Previous Swiss studies have not addressed this aspect, and our findings provide new insights into the relationship between production zones and culling risk. Production zone and alpine pasturing represent distinct factors, as animals from all zones may be sent to alpine pastures. The few differences observed between the lowlands/hilly areas and mountain 1 zones seem plausible, as the environmental impact may not be particularly significant. However, the increased culling risk for BS and OB in higher mountain zones, contrasted with the decreased risk for SF and SI, and is more difficult to explain. Given that OB is considered well-adapted to mountainous regions, we would have expected a lower culling risk for this breed in mountainous zones. Further research is needed to explore the underlying factors contributing to these unexpected results.

Alpine grazing has been linked to enhanced longevity and health in ruminants (Künzi et al., 1988, Ruhland et al., 1999). In our study, alpine pasturing was associated with a reduced culling risk across all breeds. This finding aligns with Vukasinovic et al. (2001), who also reported a significantly lower culling risk for cows sent to alpine pastures during summer. The authors attributed this to the positive influence of alpine grazing on fitness, citing the benefits of species-rich alpine pastures, fresh air and exercise, and thus longevity. However, they also acknowledge the potential bias introduced by pre-selecting only fit, pregnant and not lame cows for alpine pasturing. We agree with this interpretation and emphasize that cows sent to alpine pastures are likely preselected for being healthy and robust animals. This selection process may exclude less healthy cows and thereby inflate the apparent protective association between alpine pasturing and culling risk.

### 4.6 Farm changes, origin of replacement heifers and breed type used for insemination in heifers

We found no previous studies examining the relationship between culling risk and factors such as farm changes, the origin of heifers or insemination management. However, our findings suggest that a change of farm between the first and last lactation is associated with an increased culling risk. This may be explained by the tendency to sell lower-performing cows.

Interestingly, purchased heifers were associated with a lower risk of culling compared to those raised on the same farm. This could be because purchased animals are often bought to improve herd quality and may be retained longer due to their clear monetary value. In contrast, home-raised heifers may not be consciously valued in the same way by farmers.

In terms of insemination management, our results showed that in dairy breeds (BS, HO_HOS, HO_SHB, SF), insemination with beef or dual-purpose breeds often resulted in a slightly lower or similar culling risk compared to a dairy breed insemination. Conversely, in dual-purpose breeds (OB, SI), insemination with dairy breeds did not significantly reduce culling risk and, in some cases, increased it slightly. This slight increase may reflect an attempt to improve milk yield in the offspring of the respective cow. Supporting this, Frei (2023) found that a 10 % increase in the proportion of beef semen used increased herd life by 10 (BS) to 22 (SF) days in cows, while the effect in heifers was smaller, with an increase of 4 (BS) to 7 (OB) days.

### 4.7 Age at first calving

Our findings indicate that early calving contributes to improved survival and longevity in some dairy breeds (e.g., BS, SI) but exhibits breed-specific risk ratio patterns in others, particularly in OB and HO_HOS. Delayed AFC, particularly beyond 35 months, was associated with higher culling risks in several breeds such as BS, HO_SHB, SF, and SI.

This aligns with Bielfeldt et al. (2006), who reported a tendency for increased culling risk in Brown Swiss dairy heifers when calving occurred after three years of age. Similarly, Vukasinovic et al. (2001) observed a higher culling risk for cows with increased AFC, although they noted that the overall influence of this trait on productive lifespan was small. Interestingly, these authors found an almost linear increase in culling risk for Brown Swiss but noted a curvilinear risk pattern for Simmental and Holstein. Sewalem et al. (2005) also reported that heifers calving between 24 and 28 months had a lower risk of culling compared to older heifers across the three breeds Holstein, Jersey, and Ayrshire.

Our findings further suggest that the impact of AFC on culling risk is breed-specific and cannot be generalized. This variability likely depends on both rearing intensity and breed-specific factors. Breeds traditionally reared in alpine regions, such as OB and SI, tend to have a higher proportion of heifers with higher AFC. The seasonality of calving caused by alpine pasturing leads to a higher AFC in the later-maturing breeds. This trend is also observed in BS, though less pronounced. Additionally, these breeds had a higher proportion of animals with alpine pasturing across lactations.

### 4.8 Conformation traits and final linear score

Conformation traits appear to have limited predictive power for longevity, as evidenced by their generally low power in explaining the variation in productive lifespan. However, our findings suggest that these traits may be relatively more relevant for predicting productive lifespan in SI and OB breeds, which showed the highest explained variation for linear composite traits, albeit still at a very low level. Among the linear conformation traits, udder score had the greatest impact on culling risk, a result consistent with previous studies linking udder conformation traits to longevity (e.g., Buenger et al., 2001; Larroque & Ducrocq, 2001; Lavrinovič et al., 2009; Sawa et al., 2013; Török et al., 2021).

Similarly, the linear decrease in culling risk with an improved final linear score has been consistently reported (e.g., Hu et al., 2021; Kern et al., 2018; Sawa et al., 2013). Beyond longevity, both the overall score and the udder score have also been associated with lifetime performance (Sawa et al., 2013), highlighting the importance of functional traits, particularly udder conformation, in improving survival probability.

Despite these associations, the overall low explanatory power of linear conformation traits indicates that they represent only minor determinants of failure risk, as consistently reported in previous studies (e.g., Kern et al., 2018; Sawa et al., 2013). In contrast, fertility and production traits emerge as substantially more influential drivers of longevity, emphasizing that while conformation contributes to survival probability, reproductive efficiency and productive performance play a dominant role in culling decisions.

From a management perspective, the strong influence of fertility and production traits on culling risk highlights the importance of early detection and intervention at the herd level. Precision livestock farming tools, such as sensor-based monitoring of reproductive status and feeding behaviour, offer promising opportunities in this regard (e.g., Cavallini et al., 2025; Giannone et al., 2025). In combination with favourable environment provision (including feeding management), these approaches underline that effective herd management, supported by data-driven technologies, is key to improving dairy cow longevity (De Vries & Marcondes, 2020; Schuster et al., 2020).

## 5 Conclusions

This study investigated the impact of traits related to production, fertility, health, herd management, and conformation scores on longevity of Swiss dairy cows. The results demonstrated that most traits significantly influenced longevity, although their individual contributions to culling risk were generally small. Among the traits analyzed, fertility, as measured by the number of inseminations, had the strongest impact on culling risk, followed by the fat-protein yield relative to the cow’s herdmates. These findings underscore the importance of fertility management as a cornerstone for promoting long-lived cows, as well as the economic significance of productivity in determining dairy cow survival.

Additionally, functional cows with good udder scores and high overall linear conformation scores showed higher survival probabilities. However, the generally small contribution of individual traits to longevity highlights the complex interplay of multiple factors influencing dairy cow survival, where many traits contribute modestly despite being statistically significant. This complexity suggests that a holistic approach to herd management, encompassing fertility, productivity, health, and conformation, is essential for improving the productive lifespan of dairy cows. In this context, emerging data-driven and precision livestock farming approaches may further support early detection and management of key risk factors, thereby contributing to improved longevity.

## Funding sources

This study was conducted as part of the project “Increasing the productive lifespan of Swiss dairy cows: influencing factors, future scenarios and strategy development”, primarily financed by the Swiss Federal Office for Agriculture (OFAG, contract number: 627001582, Bern, Switzerland) with additional financial support from the Association of Swiss Cattle Breeders (ASR, Zollikofen, Switzerland), Bio Suisse (Basel, Switzerland), Fondation Sur-la-Croix (Basel, Switzerland), IP-Suisse (Zollikofen, Switzerland), Migros (Zürich, Switzerland), and Schweizer Milchproduzenten Swissmilk (SMP, Bern, Switzerland).

## Data Availability

The data used in this study are owned by the Swiss breeding organizations and managed by Qualitas AG (Zug, Switzerland). Data is available upon reasonable request under data use agreements.

## Ethical approval

This study was based exclusively on previously recorded herdbook data. No animals were handled, sampled, or subjected to any experimental procedures. Therefore, approval by an institutional or national ethics committee was not required.

## CRediT authorship contribution statement

**Anna Bieber:** Writing – original draft, Methodology, Investigation, Funding acquisition, Formal analysis, Data curation, Conceptualization. **Florian Hediger:** Methodology, Investigation, Formal analysis, Data curation. **Catherine Pfeifer:** Writing – review & editing, Data curation. **Urs Schnyder:** Writing – review & editing, Validation, Methodology, Data curation. **Florian Leiber:** Writing – review & editing, Validation, Funding acquisition. **Michael Walkenhorst:** Writing – review & editing, Project administration, Investigation, Funding acquisition, Conceptualization.

## Declaration of competing interest

The authors declare that they have no known competing financial interests or personal relationships that could have appeared to influence the work reported in this paper.

## Appendix Supplementary materials

Image, application 1

## Footnotes

Supplementary material associated with this article can be found, in the online version, at doi:10.1016/j.vas.2026.100701.
