# Supplementary material for: Effects of production, fertility, health, management and conformation scores on longevity in six Swiss dairy breeds
Source: Vet Anim Sci. 2026 May 17;33:100701. doi: 10.1016/j.vas.2026.100701 (PMC13264184; doi:10.1016/j.vas.2026.100701)
Supplement: Supplementary file 1 [file mmc1.pdf]

Supplementary material

Effects of production, fertility traits, health indicators, herd management traits and conformation scores on longevity in six Swiss dairy breeds

Anna Bieber [a], Florian Hediger [b], Catherine Pfeifer [b], Urs Schnyder [c], Florian Leiber [a], Michael Walkenhorst [a]

[a] Department of Livestock Sciences, Research Institute of Organic Agriculture FiBL, Ackerstrasse 113, 5070 Frick, Switzerland, anna.bieber@fibl.org, florian.leiber@fibl.org, michael.walkenhorst@fibl.org

[b] Department of Food System Sciences, Research Institute of Organic Agriculture FiBL, Ackerstrasse 113, 5070 Frick, Switzerland, florian.hediger@fibl.org, catherine.pfeifer@fibl.org

[c] Qualitas AG, Chamerstrasse 56, 6300 Zug, Switzerland, urs.schnyder@qualitasag.ch

Corresponding author: Anna Bieber, anna.bieber@fibl.org

Table S1. Overview on conformation traits in Brown Swiss (BS) and Original Braunvieh (OB) cows (August 2020)

Trait/ **composite trait**	Weights (%)[1]	Score 1	Score 9	Mean value (= Score 5)	Deviation 1 Score	Ideal value
**Frame**	**25**					
Stature	20			BS 147.2 cm<br>OB 142.2 cm		
Body depth	28			BS 81.7 cm<br>OB 80.3 cm		BS 85 cm<br>OB 84 cm
Chest width	28	narrow	wide			8
Top line	24	lowered	arched	1.5 cm	plane, Score 6	7
**Rump**	**10**					
Rump length	20			BS 54.8 cm<br>OB 54.2 cm		BS 58 cm<br>OB 58 cm
Rump width	20			BS 35.2 cm<br>OB 37.8 cm		BS 38 cm<br>OB 39 cm
Rump angle	40	high pins	sloped	2cm		5
Thurl position	20	rear	front	5 cm behind centre		7
**Feet & legs**	**25**					
Rear Legs Set	32	straight	sickled	150°		5
Hock development	16	a lot of fluid	clean & dry			9
Pastern joint	24	too much angle	too straight			6
Foot angle	28	low	steep	2.5 cm	0.5 cm	8
**Udder**	**30**					
Fore udder length	10	little	much	24 cm	1.5 cm	8
Fore udder attachment	14	loose	strong			9
Rear udder width	10	narrow	wide	17 cm	1.5 cm	9
Rear udder height	13	low	high			9
Udder depth	27	deep	shallow	13 cm	3 cm	7
Udder floor	13	stepped	raised	even		5
Central ligament	13	not visible	deep cleft			7
**Teats**	**10**					
Teat length	20	short	long	5 cm	1 cm	5
Teat thickness	10	thin	thick	2.5 cm		5
Teat placement	20	outside of quarter	inside of quarter	slightly inside		5
Front teat placement	30	wide	narrow	slightly outside		6
Rear teat placement	20	wide	narrow	slightly inside		4

[1] For the linear composite traits (printed in bold letters) weights refer to calculation of the final score, while for the individual conformation traits (printed in plane), these are the weights of each single trait included in the respective linear composite trait

Table S2. Overview on conformation traits in Swiss Fleckvieh (SF) and Simmental (SI) cows (version 09/2020)

Trait/ **Composite trait**[1]	Weights (%)[2]	Score 1	Score 9	Ideal value
**Frame & Capacity**	**35**			
Stature		small	tall	5
Chest width		narrow	wide	8
Body depth		shallow	deep	7-8
Muscularity		poor	strong	SF 7, SI 7-8
Expression		much	little	9
Rump angle		high pins	sloped	5
Rump width		narrow	wide	9
**Feet & legs**	**25**			
Bone structure		broad & thick	flat	8
Rear legs set		straight	sickled	5
Rear legs rear view		hock in	parallel	9
Locomotion		stiff	moveable	9
Foot angle		low	steep	7
Heel height		flat	high	7
**Udder**	**30**			
Fore udder attachment		loose	strong	9
Fore udder length		short	long	8
Rear udder height		low	high	9
Rear udder width		narrow	wide	9
Udder depth		deep	shallow	6
Central ligament		weak	strong	9
Glandularity		fleshy	soft	9
**Teats**	**10**			
Front teat length		short	long	6
Front teat placement		wide	narrow	6
Rear teat placement		wide	narrow	5

[1] Composite traits are printed in bold letters, single traits in plane letters, [2] weights used to calculate the final score

Table S3. Overview on conformation traits in Holstein cows (version 09/2020)

Trait/ **composite trait**[1]	Weights (%)[2]	Score 1	Score 9	Ideal value
**Frame & Capacity**	**25**			
Height at the top of the spine		small	tall	5
Relative height[3]		low	high	7
Chest width		narrow	wide	7
Body depth		shallow	deep	7-8
Loin (1/2)		poor	strong	9
Milk character		little	much	9
**Rump**	**10**			
Rump angle		high pins	sloped	5
Rump width		narrow	wide	9
Loin (1/2)		poor	strong	9
**Feet & legs**	**25**			
Bone structure		broad & thick	flat	8
Rear legs set		straight	sickled	5
Rear legs rear view		hock in	parallel	9
Locomotion		stiff	moveable	9
Foot angle		low	steep	7
Height of rear end of the claw		flat	high	7
**Udder**	**40**			
Fore udder attachment		loose	strong	9
Rear udder height		low	high	9
Rear udder width		narrow	wide	9
Udder depth		deep	shallow	6
Central ligament		weak	strong	8-9
Glandularity		fleshy	elastic	9
Front teat length		short	long	5
Front teat placement		wide	narrow	6
Rear teat placement		wide	narrow	5

[1] Composite traits are printed in bold letters, single traits in plane letters, [2] weights used to calculate the final score, [3] Relative height: difference between height at withers and height at the top of the spine

Table S4. Relative risk of culling for the fertility-related trait calving ease (CALV) in six Swiss dairy breeds

Classes[1]	Relative risk (RR) of culling by breed[2] and number of uncensored failures (n)											
	BS		OB		HO_HOS		HO_SHB		SF		SI	
	RR	n	RR	n	RR	n	RR	n	RR	n	RR	n
unassisted[3]	1.00	312,442	1.00	11,176	1.00	76,248	1.00	128,152	1.00	149,723	1.00	29,436
light help	1.08****	249,292	1.00	11,114	1.00	70,891	1.03****	128,876	1.01***	137,309	1.00	33,550
difficult	1.06****	27,561	1.47****	1480	1.00	9455	1.03**	18,484	1.01	16,887	1.02	2888
Caesarean section	1.01	1393	1.08	142	1.00	374	1.00	864	1.00	988	1.01	303
NA	0.85****	116,909	0.77****	4544	1.00	88,465	0.92****	97,541	0.96****	174,876	0.90	21,541

[1] NA= missing values; [2]Breed: BS= Brown Swiss, OB= Original Braunvieh, HO_HOS= Holstein from the Holstein Switzerland herdbook, HO_SHB= Holstein from the Swissherdbook, SF= Swiss Fleckvieh, SI= Simmental; [3] reference class; risk levels differ significantly from the reference class according to the Chi-square statistic as follows: **P ≤ 0.01, ***P ≤ 0.001, ****P ≤ 0.0001. RR > 1.0 indicates an increased culling risk compared to the reference, while RR < 0.1 indicates a reduced culling risk.

Table S5. Relative risk of culling for herd size (HS) and relative herd size changes (RHS) in six Swiss dairy breeds

Traits and classes[1]	Relative risk (RR) of culling by breed[2] and number of uncensored failures (n)											
	BS		OB		HO_HOS		HO_SHB		SF		SI	
	RR	n	RR	n	RR	n	RR	n	RR	n	RR	n
HS												
<10	0.84****	72,445	0.89****	5450	1.00	52,364	0.59****	17,521	0.89****	45,387	0.91****	20,735
10-19	1.02****	244,897	1.05***	11,146	0.81****	35,187	0.98****	75,393	1.04****	182,874	1.00	38,543
20-39[3]	1.00	293,466	1.00	9881	1.03***	35,345	1.00	170,265	1.00	196,222	1.00	22,175
40-69	0.96****	82,250	1.00	1761	1.12****	18,075	0.91****	79,642	0.89****	44,381	0.96*	4140
≥70	0.90****	14,539	0.80**	218	1.93****	104,462	0.89****	31,096	0.87***	10,919	1.02	2125
RHS												
shrinking	0.87****	85,175	0.69****	3480	1.08****	34,507	0.76****	36,133	0.89****	53,137	0.88****	10,201
constant[3]	1.00	527,910	1.00	21,002	1.00	185,024	1.00	294,686	1.00	360,991	1.00	66,121
increasing	0.98****	94,512	1.01	3974	0.95****	25,902	0.97****	43,098	0.97****	65,655	1.00	11,396

[1] Class distribution: RHS (relative herd size changes): shrinking :> -20%, constant: between -20 and +20% change, increasing: > + 20 % , [2]Breed: BS= Brown Swiss, OB= Original Braunvieh, HO_HOS= Holstein from the Holstein Switzerland herdbook, HO_SHB= Holstein from the Swissherdbook, SF= Swiss Fleckvieh, SI= Simmental; [3] reference class; risk levels differ significantly from the reference class according to the Chi-square statistic as follows: * P ≤ 0.05, ***P ≤ 0.001, ****P ≤ 0.0001. RR > 1.0 indicates an increased culling risk compared to the reference, while RR < 0.1 indicates a reduced culling risk.

Table S6. Relative risk of culling for calving season (CS) in six Swiss dairy breeds

Classes[1]	Relative risk (RR) of culling by breed[2] and number of uncensored failures (n)											
	BS		OB		HO_HOS		HO_SHB		SF		SI	
	RR	n	RR	n	RR	RR	RR	RR	n	RR	n	RR
Winter[3]	1.00	427,235	1.00	17,437	1.00	137,066	1.00	213,900	1.00	282,824	1.00	60,984
Summer	0.96****	280,362	0.93****	11,019	0.90****	108,367	0.96****	160,017	0.93****	196,959	0.87****	26,734

[1] Class distribution: Winter: 1. September to 28./29.February, Summer: 1. March to 31. August, [2]Breed: BS= Brown Swiss, OB= Original Braunvieh, HO_HOS= Holstein from the Holstein Switzerland herdbook, HO_SHB= Holstein from the Swissherdbook, SF= Swiss Fleckvieh, SI= Simmental; [3] reference class; risk levels differ significantly from the reference class according to the Chi-square statistic as follows: ****P ≤ 0.0001. RR > 1.0 indicates an increased culling risk compared to the reference, while RR < 0.1 indicates a reduced culling risk.

Table S7. Relative risk of culling for production zone (ZONE) and alpine pasturing (ALP) in six Swiss dairy breeds

Traits and classes[1]	Relative risk (RR) of culling by breed[2] and number of uncensored failures (n)											
	BS		OB		HO_HOS		HO_SHB		SF		SI	
	RR	n	RR	n	RR	n	RR	n	RR	n	RR	n
ZONE												
lowland/hilly[3]	1.00	342,284	1.00	8173	1.00	178,145	1.00	233,447	1.00	249,876	1.00	18,454
mountain 1	1.03****	101,220	1.00	5076	0.99	47,236	1.03****	67,952	0.98****	100,672	0.91****	14,579
mountain 2	1.05****	151,296	0.99	8803	0.97**	16,657	0.96****	53,749	0.96****	88,733	0.95****	22,082
mountain 3/4	1.10****	94,805	1.02	5611	1.00	3395	1.01	10,874	0.96****	34,893	1.00	31,122
NA	0.42****	17,992	0.31****	793	-	0	0.26****	7895	0.21****	5609	0.51****	1481
ALP												
no[3]	1.00	615,046	1.00	22,379	1.00	242,246	1.00	356,421	1.00	458,731	1.00	74,838
yes	0.65****	92,261	0.65****	6076	0.94****	3187	0.76****	17,312	0.62****	21,032	0.58****	12,865
NA	0.92	290	0.81	1	-	0	1.24*	184	0.93	20	1.00	15

[1] ZONE: The Federal Office for Agriculture records the agricultural zones and areas in digital topographical maps, which can be accessed via the following link: https://s.geo.admin.ch/6ee4f215a7, NA= missing values, [2]Breed: BS= Brown Swiss, OB= Original Braunvieh, HO_HOS= Holstein from the Holstein Switzerland herdbook, HO_SHB= Holstein from the Swissherdbook, SF= Swiss Fleckvieh, SI= Simmental; [3] reference class; risk levels differ significantly from the reference class according to the Chi-square statistic as follows: * P ≤ 0.05, **P ≤ 0.01, ****P ≤ 0.0001. RR > 1.0 indicates an increased culling risk compared to the reference, while RR < 0.1 indicates a reduced culling risk.

Table S8. Relative risk of culling for occurrence of farm change between first and last lactation (CHANGE), ORIGIN of the heifer (own or purchase) in six Swiss dairy breeds

Traits and classes	Relative risk (RR) of culling by breed[1] and number of uncensored failures (n)											
	BS		OB		HO_HOS		HO_SHB		SF		SI	
	RR	n	RR	n	RR	n	RR	n	RR	n	RR	N
CHANGE												
yes[2]	1.00	656,458	1.00	26,104	1.00	234,292	1.00	330,315	1.00	427,491	1.00	77,248
no	1.25****	51,139	1.46***	2352	0.47****	11,141	1.32****	43,602	1.09****	52,292	1.25****	10,470
ORIGIN												
own[2]	1.00	568,539	1.00	21,379	1.00	204,171	1.00	295,533	1.00	394,000	1.00	69,120
purchase	0.88****	139,058	0.86****	7077	0.94****	41,262	0.92****	78,384	0.89****	85,783	0.86****	18,598

[1] Breed: BS= Brown Swiss, OB= Original Braunvieh, HO_HOS= Holstein from the Holstein Switzerland herdbook, HO_SHB= Holstein from the Swissherdbook, SF= Swiss Fleckvieh, SI= Simmental; [2] reference class; risk levels differ significantly from the reference class according to the Chi-square statistic as follows: ***P ≤ 0.001, ****P ≤ 0.0001. RR > 1.0 indicates an increased culling risk compared to the reference, while RR < 0.1 indicates a reduced culling risk.

Table S9. Relative risk of culling for breed type used for the first insemination as heifer (BREEDTYPE) in six Swiss dairy breeds

Traits and classes[1]	Relative risk (RR) of culling by breed[2] and number of uncensored failures (n)											
	BS		OB		HO_HOS		HO_SHB		SF		SI	
	RR	n	RR	n	RR	n	RR	n	RR	n	RR	n
BREEDTYPE[3]												
dairy	1.00	509,089	1.10	1427	1.00	203,216	1.00	273,923	1.00	252,609	1.09****	2637
beef	0.95****	133,828	0.94***	4324	0.99	18,997	0.92****	53,348	0.88****	89,698	0.97*	6927
dual-purpose	0.93****	16,512	1.00	21,253	1.00	642	0.95**	3298	0.90****	24,767	1.00	64,763
other	0.92*	804	0.92	24	0.97	4105	0.94	310	0.93	153	0.99	11
NA	0.72****	47,364	0.87****	1428	0.89****	18,473	0.81****	43,038	0.79****	112,556	0.83****	13,380

[1] NA= missing values, [2]Breed: BS= Brown Swiss, OB= Original Braunvieh, HO_HOS= Holstein from the Holstein Switzerland herdbook, HO_SHB= Holstein from the Swissherdbook, SF= Swiss Fleckvieh, SI= Simmental; [3] reference class is the one with RR= 1.00 and differs by breed; risk levels differ significantly from the reference class according to the Chi-square statistic as follows: * P ≤ 0.05, **P ≤ 0.01, ****P ≤ 0.0001. RR > 1.0 indicates an increased culling risk compared to the reference, while RR < 0.1 indicates a reduced culling risk.

Table S10. Relative risk of culling for age at first calving (AFC) in months in six Swiss dairy breeds

Classes	Relative risk (RR) of culling by breed[1] and number of uncensored failures (n)											
	BS		OB		HO_HOS		HO_SHB		SF		SI	
	RR	n	RR	n	RR	n	RR	n	RR	n	RR	N
<24	0.85****	5249	0.76*	53	1.02*	15,098	0.89****	15,254	0.91****	14,508	0.93*	648
24-26	0.97****	64,919	0.81****	724	0.92****	76,277	0.94****	107,889	0.95****	111,709	0.95****	9169
27-29[2]	1.00	153,691	1.00	3454	1.00	69,787	1.00	110,034	1.00	134,955	1.00	17,312
30-32	1.02****	182,083	0.89****	7924	0.96****	44,644	0.98***	74,437	0.99*	110,648	1.03**	22,123
33-35	1.04****	129,168	0.97	6912	0.97***	19,710	1.01*	33,793	1.02***	55,621	1.05****	17,792
>35	1.08****	172,487	0.99	9389	0.99	19,917	1.03***	32,510	1.03***	52,342	1.04****	20,674

[1]Breed: BS= Brown Swiss, OB= Original Braunvieh, HO_HOS= Holstein from the Holstein Switzerland herdbook, HO_SHB= Holstein from the Swissherdbook, SF= Swiss Fleckvieh, SI= Simmental; [2] reference class; risk levels differ significantly from the reference class according to the Chi-square statistic as follows: * P ≤ 0.05, **P ≤ 0.01, ***P≤ 0.001, ****P ≤ 0.0001. RR > 1.0 indicates an increased culling risk compared to the reference, while RR < 0.1 indicates a reduced culling risk.
